# Why Is Aneuploidy Associated with Favorable Outcome in Neuroblastoma?

**DOI:** 10.3390/biom11081116

**Published:** 2021-07-29

**Authors:** Gian Paolo Tonini

**Affiliations:** Laboratory of Target Discovery and Biology of Neuroblastoma, Pediatric Research Institute, Città della Speranza, Corso Stati Uniti 4, 35127 Padova, Italy; gp.tonini@irpcds.org

**Keywords:** neuroblastoma, tumorigenesis, chromosome instability, aneuploidy

## Abstract

Neuroblastoma is a pediatric cancer, onset with localized as well as metastatic disease. Localized tumors usually show a high content of aneuploid cells. It is suggested that aneuploid cells with numerical copy number variation (CNV) are generated by chromosome instability (CIN). Patients with a localized tumor respond well to the therapy and show a good outcome. On the contrary, patients with a metastatic tumor have worse outcomes and the cells with structural CNV show high levels of CIN. It is proposed that a favorable outcome in patients with localized disease is associated to the grade of CIN.

Neuroblastoma is a pediatric cancer showing great biological and clinical heterogeneity. The prognosis of neuroblastoma patients ranges from favorable to severe outcomes. Moreover, in about 70% of patients younger than one year of age, the tumor may undergo spontaneous or drug-induced regression [1,2]. This peculiar behavior of the neuroblastoma has not yet been solved.

The advent of new technologies such as an array comparative genomic hybridization (aCGH) [3], a genome wide association study (GWAS) [4], and whole-exome sequencing (WES) [5], have allowed us to clarify some aspects of different outcome in neuroblastoma patients [6].

In a normal situation, each cell has two copies of chromosomes and they are designated diploid cells. Localized tumors of patients under one year of age are usually characterized by aneuploidy, a numeric whole extra-chromosome copy number with or without structural abnormalities [1]. Aneuploidy is defined as the presence of an unbalanced number of chromosomes or large portions of chromosomes in a cell (Figure 1) [7].

How is generating the whole extra-chromosome numbers is not yet fully understood. It is generally accepted that they are produced by the malfunction of mitosis apparatus; for example, the failure of spindle bodies, resulting in an unequal chromosome number distribution in the daughter cells.

There are very few data about the activity of extra-copy chromosomes in neuroblastoma cells, but there are several indirect pieces of evidence that extra-chromosomes partially contribute to the tumor aggressiveness [8]. For instance, cells of localized tumors are not able to grow in vitro and in animals. As a consequence, it is plausible that aneuploid neuroblastoma cells have low aggressiveness, and the extra-chromosomes give a partial contribution to the tumor aggressiveness. Tumor cells of patients in stage 1 or 2 or 4S [9] are characterized by high aneuploidy, indicating that aneuploidy status is associated to tumors in patients with a good prognosis. On the contrary, neuroblastoma cells of stage 4 have gross structural chromosome damages including chromosome deletion, chromosome gain, and chromosome rearrangement, and they are very aggressive [9]. These structural chromosome variations were prevalent observed in the advanced clinical stage of patients with severe diagnosis. We have shown that neuroblastoma cells of metastatic stage 4 in patients older than 18 months have a higher percentage of structural chromosome copy number variations. It is possible that structural chromosome aberrations damage several genes.

The aneuploidy is characterized by chromosome instability (CIN). CIN is a complex phenomenon that mainly includes the chromosome mis-segregation [10,11,12,13,14]. It is possible that the aggressiveness of neuroblastoma cells depends on the degree of CIN.

It is difficult to define the degree of CIN, but many genes are involved in CIN: genes regulating mitosis and DNA repair genes have been reported with different methods for the assessment of chromosomal changes in solid cancer. The methods include different techniques to explore the CIN: interphase-FISH, flow cytometry, SNParray, Micronuclei counting, CGH array, digital PCR, and karyotyping. Carter et al. [15] have found a CIN gene signature for many cancers including neuroblastoma [16]. In view of the foregoing, the evaluation of degree of CIN appears very complex and many parameters have to be considered. All of the above information suggests the following assumption. The aggressiveness of the tumor depends mainly on the degree of CIN that generates aneuploidy and the grade of aneuploidy is associated with favorable outcomes in neuroblastoma.

This aspect should be taken into consideration when we include CIN-related drugs in the neuroblastoma therapy. [5].

## Figures and Tables

**Figure 1:**
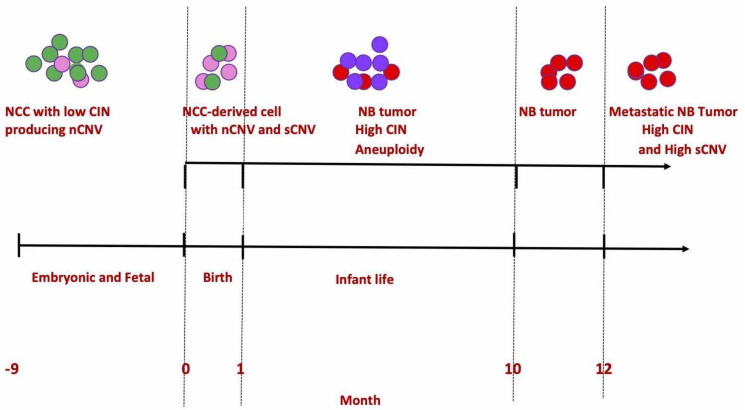
In the figure is a schematic illustrated the hypothesis of the formation of aneuploidy neuroblastoma cells. It is widely accepted that neuroblastoma origins from Neural Crest Cells (NCC). NCC (green circle) are a group of neural crest cells in which the CIN is very low and present in few cells (pink circle). After birth of the baby, the number of cells (blue circle) with chromosome extra-copies increases. The cells have a high numerical Copy Number Variation (nCNV) and form aneuploid cells of tumors of favorable disease. After the first year of life the tumor cells accumulate several structural Copy Number Variations (sCNV), producing aggressive neuroblastoma cells (red circle).

## Data Availability

Not applicable.
